# A rapid pre-implementation evaluation to inform a family engagement navigator program during COVID-19

**DOI:** 10.1186/s43058-020-00098-2

**Published:** 2020-12-09

**Authors:** Stephanie Parks Taylor, Robert T. Short, Anthony M. Asher, Brice Taylor, Rinad S. Beidas

**Affiliations:** 1 Department of Internal Medicine, Atrium Health’s Carolinas Medical Center, 1000 Blythe Blvd. MEB 5th Floor, Charlotte, NC 28203 USA; 2 University of North Carolina School of Medicine, Chapel Hill, USA; 3 Department of Internal Medicine, Division of Pulmonary and Critical Care, Atrium Health’s Carolinas Medical Center, Charlotte, USA; 4 Department of Psychiatry, Perelman School of Medicine, University of Pennsylvania, Philadelphia, USA; 5 Department of Medical Ethics & Health Policy, Perelman School of Medicine, University of Pennsylvania, Philadelphia, USA; 6 Department of Medicine, Perelman School of Medicine, University of Pennsylvania, Philadelphia, USA; 7 Penn Implementation Science Center at the Leonard Davis Institute of Health Economics (PISCE@LDI), University of Pennsylvania, Philadelphia, USA

**Keywords:** COVID-19, Family-centered care, Critical care, Family engagement, Rapid analysis

## Abstract

**Background:**

Innovative models of family engagement and support are needed in the intensive care unit (ICU) during times of restricted visitation such as the COVID-19 pandemic. Limited understanding of the factors affecting the uptake and outcomes of different family support models hinders the implementation of best practices. We aimed to conduct a rapid pre-implementation evaluation of stakeholder-perceived facilitators and barriers to design implementation strategies to support a novel program using medical students to facilitate family-centered care in the ICU.

**Methods:**

We conducted a 2-step process. In step 1, we gathered contextual data via interview-style open-ended questions sent to clinicians and navigator stakeholders via email. We used electronic data collection due to the physical distancing requirements, the need to prioritize brief data collection for respondent burden, and the need for rapid knowledge gain. A codebook based on the Consolidated Framework for Implementation Research (CFIR), an integrated framework from the field of implementation science, was used to analyze the findings. In step 2, a pilot of the intervention was implemented with 3 navigators over 2 weeks. Implementation strategies were developed to target barriers identified by the pre-implementation evaluation.

**Results:**

Fourteen (70%) of the identified stakeholders responded to the survey. Ten constructs encompassing all five CFIR domains were present in responses as implementation influencers, with the Intervention domain most frequently represented. Through these results and operational feedback from navigators during the pilot period, stakeholders selected multiple implementation strategies: audit and provide feedback, develop educational materials, conduct ongoing training, promote adaptability, assess and redesign workflow, identify and prepare champions, and engage community resources.

**Conclusions:**

We demonstrated how a conceptually based pre-implementation program evaluation can be used to rapidly inform optimal implementation strategies. We report key factors to inform design and implementation strategies for a novel ICU family engagement navigator program that may be useful to others wishing to adopt similar programs.

**Supplementary Information:**

The online version contains supplementary material available at 10.1186/s43058-020-00098-2.

Contributions to the literature

- Family engagement is a key component of high-quality care for critically ill patients, but restricted visitation policies due to COVID-19 challenge the delivery of family-centered care.
- We conducted a formative evaluation to rapidly inform the design and implementation of a medical student-led multicomponent family engagement navigator program.
- Our results provide actionable facilitators and barriers for other organizations seeking to implement similar programs.
- Our study demonstrates successful application of a novel rapid methodologic approach that allowed efficient knowledge gain to inform program design under the time pressure generated by the COVID-19 pandemic.

## Background

Family-centered care, the inclusion of family members as part of a collaborative team including patients, families, and healthcare teams, is recognized as a key component of high-quality ICU care [1]. However, restricted family presence due to physical distancing requirements associated with the COVID-19 pandemic challenges the provision of family-centered care [2], with some centers describing family communication as “complex and fragmented.” [3]. A Society of Critical Care Medicine position statement provided recommendations for optimal family-centered care during an outbreak setting including (i) assess family coping, (ii) increase family communication and support, and (iii) guide families regarding possible engagement strategies during a crisis [4]. Novel implementation strategies for delivering family-centered care during times of restricted visitation are urgently needed [2]. One strategy involves leveraging skills of persons outside of the clinical team such as medical students, social workers, or providers in specialties other than critical care as “communication navigators” to provide additional support to families [2, 5]. This approach is supported by evidence for the success of communication facilitators in other settings [6, 7], and using medical students in this role may have reciprocal benefits in terms of educational and experiential impact. Unfortunately, factors affecting the adoption and success of differing models of delivery in specific health settings are not well characterized.

The application of implementation science, a field focused on promoting the uptake of evidence-based practices, may provide new insights into the development of new family engagement and support models. Implementation science leaders have called for innovations in methodology that enhance the pace of knowledge translation [8, 9]. In this study, we used a conceptually grounded pre-implementation evaluation to rapidly inform the design of a new program using medical students to facilitate family engagement when families cannot be at the bedside due to restrictions on visitation due to the COVID-19 pandemic. The objective was to assess readiness and identify facilitators and barriers to the intervention which were used to develop contextually appropriate implementation strategies. Our analyses were guided by an implementation science framework, the Consolidated Framework for Implementation Science (CFIR) [10]. All study activities were approved by the Atrium Health Institutional Review Board.

## Methods

### Setting

The pre-implementation evaluation was conducted at an urban tertiary care teaching hospital in North Carolina with over 100 ICU beds.

### Description of the intervention

Family engagement navigators are intended to fill three primary roles: (i) facilitate communication between the patient’s care team and the family member, (ii) promote humanization of the patient, and (iii) provide emotional support to the family. Using collaborative exchange with others implementing a similar program in another health system [5], we developed a general operational workflow as follows: new ICU patients are assigned to navigators on the first day of admission. Navigators identify a primary contact for the family, help the family determine the best platform for communication (phone, videoconferencing application, etc.), and assist in overcoming any barriers to accessing communication platforms, including assistance with videoconference applications and designating preferred timing of contact from the clinical team. Navigators facilitate communication as needed, such as helping families keep a list of questions to ask the clinical team. Additionally, navigators provide emotional support to families, allowing them to process their fears, anxieties, and stress related to their loved ones’ illness.

**Fig. 1:**
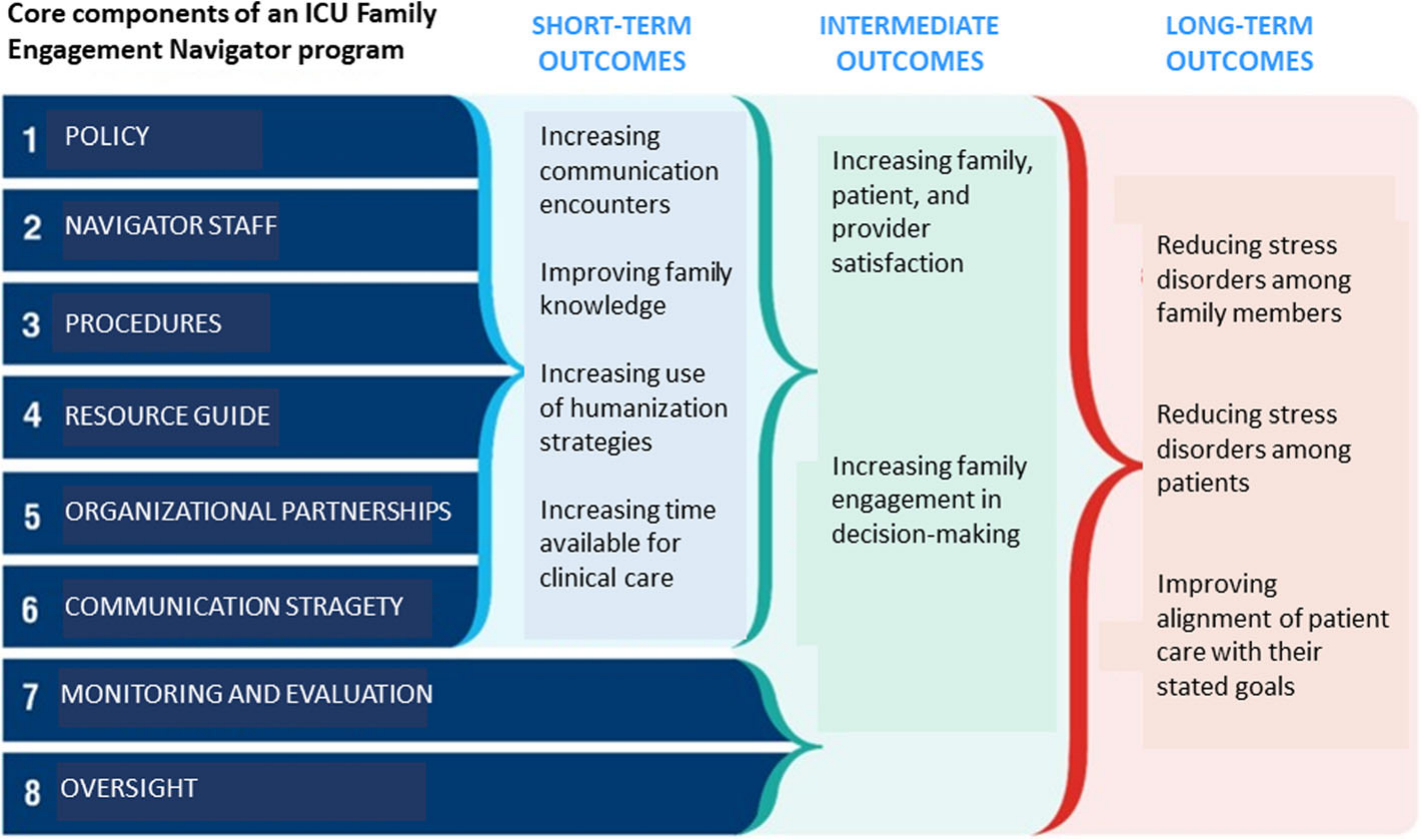
Core components for a successful family engagement navigator program and anticipated outcomes

Stakeholders including intensivists, nurses, researchers, and medical students worked together to develop a conceptual model for the program. Figure 1 outlines the eight core components identified as necessary for a successful family engagement navigator program. *Policies* set forth expectations related to what types of information can be shared by navigators and specifications for maintaining privacy. *Navigator staff* are the people providing the communication support role—in our setting, we are leveraging the skills of medical students who are removed from direct patient care for this role. *Procedures* provide standardized and concise instructions for navigators to initiate contact, respond to difficult situations, and disengage when appropriate. A *resource guide* serves as an important tool for navigators to access education materials. Strategic *organizational partnerships* between navigators and ICU teams are needed to ensure consistent and non-duplicative effort. A *communication strategy* creates awareness of the navigator program among patients, families, and ICU providers. Establishing a *monitoring and evaluation* plan and system allows for evaluating the extent to which the family engagement navigation program is achieving its intended objectives. Lastly, *oversight* ensures that the program is being implemented in a standardized manner and is on track to achieve outcomes.

This program model is intended to provide a standardized approach for implementing a family engagement navigator program to deliver family-centered care, with the short-term goals of (i) increasing communication encounters among family members, patients, and the healthcare team; (ii) improving family knowledge about the logistics of communication during their loved ones’ hospitalization; (iii) increasing the use of humanization strategies such as the “get to know me” board; and (iv) increasing the amount of time available to bedside providers for clinical care. The intermediate-term goals include (i) family, patient, and provider satisfaction with the program and (ii) increasing family engagement with decision-making. Ultimately, the desired long-term outcomes of the family engagement navigation program are (i) reduced stress disorders among family members including anxiety and post-traumatic stress disorder, (ii) reduced stress disorders among patients, and (iii) alignment of patient care with their stated goals. The Template for Intervention Description and Replication (TIDieR) checklist [11] is available in the supplementary material to provide a detailed overview of the intervention to ensure transparent reporting.

### Pre-implementation evaluation

**Fig. 2:**
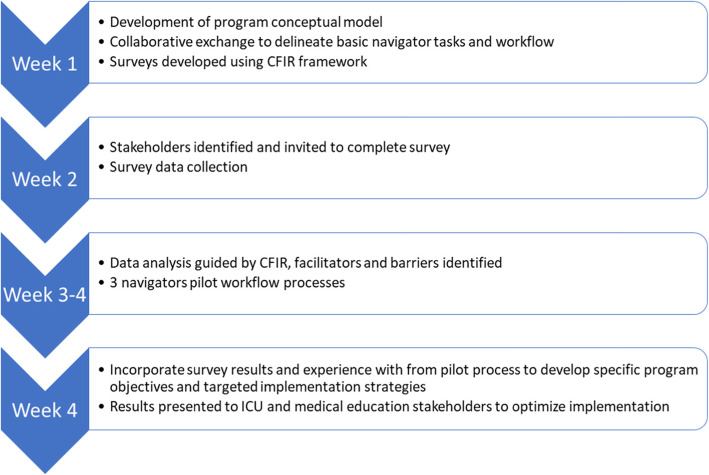
Overview of the pre-implementation evaluation timeline

We conducted a 2-step rapid pre-implementation process. In step 1, we gathered contextual data via survey sent to clinicians and navigator stakeholders via email. We used electronic data collection rather than in-person interviews due to the physical distancing requirements, the need to minimize respondent burden during the early days of the crisis, and the need for rapid knowledge gain. In step 2, a pilot of the intervention was implemented with 3 navigators over 2 weeks. An overview of the pre-implementation workflow is shown in Fig. 2.

### Participants and recruitment

In step 1, we used a purposive sampling stratified by role (physician, nurse, or navigator) to identify 20 clinicians and potential navigators who were especially knowledgeable about family engagement and likely to communicate their experiences and opinions in an expressive, reflective manner. The sampling technique was chosen to maximize the information yield and scope while efficiently using limited resources for data analysis. We sent the surveys via email in April 2020 and received responses within 2 weeks. Stakeholders included 11 clinicians (8 intensivist physicians and 3 ICU nurses), and 9 medical students signed up to be navigators. Participation was voluntary, and responses were anonymous.

### Surveys

The open-ended survey questions were designed to mimic interview style and encourage detail-rich responses using REDCap electronic data capture tools [12]. We expected respondents to be familiar with the ICU communication processes and the typical inclusion of family engagement in ICU care, and we provided a brief description of the potential role of a medical student navigator in the introduction to the survey. We used the CFIR to guide the development of the open-ended questions, using a “menu of constructs” approach which involves systematic and comprehensive exploration and identification of potential explanatory themes or factors to understand a complex intervention [13].

The CFIR provides a comprehensive taxonomy of operationally defined constructs that are likely to influence the implementation of complex programs [10]. CFIR constructs are organized into five major domains, including the characteristics of the family engagement navigator program (e.g., evidence strength and quality, adaptability), the outer setting (e.g., patient needs and resources), the inner setting (e.g., infrastructure, implementation climate, compatibility of the program with existing workflow), the process used to implement the program (e.g., engagement of key stakeholders, receipt of feedback), and the characteristics of individuals involved (e.g., knowledge and beliefs, and self-efficacy). The survey for ICU clinicians was similar to the questionnaire for navigators, except the navigator survey also included questions about self-efficacy and program training.

### Theoretical framework, coding, and analysis

We deductively analyzed responses to open-ended questions using CFIR constructs to organize and interpret respondents’ perceptions and determine the most critical pre-implementation factors likely to influence the successful adoption of the model within the ICU organizational setting. A member of the study team developed a codebook using the CFIR codebook template [14] and analysis focused on the central a priori research questions and CFIR constructs. Using a team coding approach, the lead coder and a second coder applied these categories. Both coders met regularly to review and compare the application of the codes with discussion and adjudication of any discrepancies. A third coder reviewed the final application of the coding schema to ensure consistent and comprehensive analysis. We prioritized a rapid but rigorous analysis process and continued discussion until full agreement was achieved.

### Step 2: Pilot process

During the pilot implementation, three student navigators supported 1–2 families at a time over a 2-week period. During this time, navigators attended to operational and workflow processes including time resources required to deliver family engagement support and processes for exchange of information with clinical teams. Pilot navigators kept daily logs of the process measures (e.g., family participation, number of calls, time per call, successful and failed attempts to share information with care teams, and other observations). Using contextual data obtained from step 1 and data from step 2 navigators, the study team identified specific implementation strategies that are likely to be feasible and effective organized by the Expert Recommendations for Implementing Change (ERIC) taxonomy [15].

## Results

Of the 20 stakeholders identified for the survey, 14 (70%) responded to the email invitation (8 intensivists and nurse clinicians, 6 navigators). Respondents spent an average of 10 min responding to the survey. In general, participants were enthusiastic about the program and expressed confidence that it would succeed. Respondents’ insights and feedback from the navigators during the pilot implementation fell within several relevant CFIR constructs spanning all five of the framework domains (Table 1). These barriers and facilitators are presented based on the five domains within the framework provided by the CFIR.

**Table 1:** Respondents’ insights about the family engagement navigator model categorized by the CFIR domain and construct

CFIR domain	Construct/subconstruct	Findings	Example
**Intervention**	**Intervention source:** Perception about whether the intervention is externally or internally developed	Respondents recognized that the intervention was designed by frontline stakeholders.	“It’s going to be well-received because the idea germinated from within, not from people who don’t know the details of day-to-day experience”
	**Evidence strength and quality:** Perceptions of the quality and validity of evidence supporting the belief that the intervention will have desired outcomes	The program was likened to other successful communication facilitation programs.	“Outside of COVID, there are studies that support using external team members to support family communication”
	**Adaptability**: The degree to which an intervention can be adapted, tailored, refined, or reinvented to meet local needs	The navigator program was perceived as adaptable to meet the individualized needs of families.	“We may need to vary the frequency we check in with families” “Some families may prefer certain technology than others”
	**Relative advantage**: Stakeholders’ perception of the advantage of implementing the intervention versus an alternative solution	The family engagement navigator program was preferred to RN or MD providing support because it offloads clinicians doing bedside care.	“Family support was once incorporated into bedside rounding but now requires additional time commitment that could interfere with clinical responsibilities” “It could offload some of the work for the bedside team”
	**Complexity:** Perceived difficulty of the intervention, reflected by duration, scope, radicalness, disruptiveness, centrality, and intricacy and number of steps required to implement	Respondents worried that identifying patients and exchanging information between navigators and the clinical team are not straightforward.	“The logistics of coordinating between navigators and medical team… will be the major barrier” “It could be productive, if structured correctly”
**Outer setting**	**Patient needs and resources**: The extent to which patient needs, as well as barriers and facilitators to meet those needs, are accurately known and prioritized by the organization	Respondents were concerned that access to technology and language barriers could impede equitable implementation of the intervention.	“There are a lot of factors we cannot control (language barriers, reliable phone connection/access)”
		Some clinicians feared that families would be frustrated if navigators could not give clinical updates.	“Families may not be sure what the aim is, since they can’t convey information about prognosis, status, or plan of care” “Most families will just want to know about the medical updates”
	**Culture**: Norms, values, and basic assumptions of a given organization	Respondents perceived that family engagement was a priority for the organization and that new ideas are typically embraced by the organization.	“We’re really focused on family engagement, and this will help to get back to that” “New ideas are embraced, especially in the COVID era”
	**Implementation climate**:		
	• *Tension for change*: The degree to which stakeholders perceive the current situation as intolerable or needing change	Most respondents strongly endorsed a need for solution to family engagement and support during restricted visitation.	“Communication with families is necessary and creates a better experience for everyone” “I would be terrified if I couldn’t visit my loved one in the ICU” “It has been very difficult to communicate with families”
	• *Compatibility*: How the intervention fits with existing workflows and systems	Clinicians felt the program could easily fit into the existing workflow but worried about role confusion and inconsistent information.	“We must ensure that redundancies in communication are minimized” “It will require collaboration so that information remains consistent”
		Respondents expressed a strong opinion that this intervention be additive not substitutive for communication with clinical team.	“It will be helpful but won’t replace the role of a team member actually caring for the patient calling”
**Individuals**	**Knowledge and beliefs:** Individuals’ attitudes toward and value placed on the intervention as well as familiarity with facts, truths, and principles related to the intervention	Respondents placed a high value on family engagement and were enthusiastic about the family engagement navigator program’s success.	“Communication with families is necessary and creates a better experience for everyone” “I think this will improve communication between patients, families, and treatment teams” “This will help families feel some sort of control by empowering them to ask questions”
		Clinicians requested more information on the workflow and role definitions.	“I would like to know more about how it will work, what the navigator will do versus what I will do”
	**Self-efficacy:** Individual belief in their own capabilities to execute courses of action to achieve implementation goals	Navigators felt confident in their ability to provide support to ICU families. Medical students felt that the training and available resources were effective.	“I am very confident that we can successfully implement the program” “The resources are helpful in building skills for communication, empathy, and team-based care”
**Process**	**Engagement:** Attracting and involving appropriate individuals in the implementation and use of the intervention through a combined strategy of social marketing, education, role modeling, training, and other similar activities	Both navigators and clinicians could readily identify “champions” (people in the organization who are likely to go above and beyond what might be expected) for the intervention.	“I could definitely see (Provider A or Provider B) taking this on!”
	**Reflecting/evaluation:** feedback about the progress and quality of implementation accompanied with regular personal and team debriefing about progress and experience	Navigators placed importance on receiving feedback from families and others about the impact of intervention.	“I would like to get family feedback to see if it was helpful” “I would like to know what the medical team thinks of the communication strategy”

*CFIR* Consolidated Framework for Implementation Research [10]

### Domain 1: Intervention characteristics

Respondents recognized that the family engagement navigator model was designed by frontline stakeholders with support from the project leads and backed by administrative support in the medical school. Respondents felt that buy-in and uptake would be facilitated because it was perceived as a “bottom-up” model rather than a “top-down” imposed initiative.

In addition to intervention source, other key constructs of an intervention identified by the CFIR include the strength of evidence supporting the intervention and its perceived relative advantage over the interventions in use. In this project, one respondent mentioned literature supporting the success of similar ICU communication facilitator models [6, 7], and several respondents reported strong evidence supporting improved outcomes associated with family engagement in the ICU [1, 2]. Many clinician respondents perceived the family engagement navigator program to have a relative advantage over alternative solutions, such as the bedside nurse or clinical team providing this support, because of the already high workloads for these clinicians: “Family support was once incorporated into bedside rounding but now requires additional time commitment that interfere with clinical responsibilities,” “Sometimes tracking down the right family member and contact can be time consuming… It could offload some of the work for the bedside team.”

Adaptability of the model was identified as a strength by respondents, who recognized that the model comprised core components (communication facilitation, humanization, and emotional support) and adaptable components (other types of support). Nearly all respondents identified patient needs and resources as a source of potential barriers to the program’s success. Specific barriers in this category included access and familiarity with technology: “Any barriers to electronic communication will be obstacles for the intervention: lack of wifi, lack of reliable phone, etc.” Respondents also frequently identified language barriers: “Some of us [navigators] are fluent in Spanish but it will be difficult for families that speak other languages.” Respondents noted that increased demands on family members’ time may limit their availability to communicate: “Availability to talk [might be a barrier], in terms of their occupation and other things they have to do.” Thus, respondents noted that the adaptability of the program was a facilitator of overcoming these barriers, as it allowed for the implementation of the model to families with variable needs and different combinations of support provided.

Another CFIR intervention construct, complexity, can be a barrier to effective implementation. Respondents identified that the process of providing family support process is well-defined, but the process of identifying new patients and exchanging information between navigators and the clinical team may require multiple steps and is not straightforward. From the pilot process, navigators reported that supporting family engagement required multiple calls each day, comprising an average of 1 h per day per family. Respondents and navigators provided potential solutions to this barrier such as creating medical record note templates (Supplemental File) and shared documents for the clinical team and navigator to efficiently exchange transmission.

### Domain 2: Outer setting

Respondents recognized an international initiative to enhance family engagement for ICU patients [1] and felt that the family engagement navigator program was aligned well with this external directive, facilitating buy-in, participation, and uptake of the model. Respondents indicated that the implementation of the family engagement navigator was facilitated by the high degree of existing social capital and relationships between the medical student community and the clinical ICU teams. The ICU clinicians had experience working with medical students when they were involved in direct clinical care, and this facilitated trust in the navigators to successfully provide support to families. High social capital and networked relationships are associated with the efficiency of implementation [16], suggesting that this will be a robust outer setting facilitator for the family engagement navigator program.

### Domain 3: Inner setting

Two subconstructs of implementation climate, a construct in the inner setting domain, were identified as key contextual factors for program implementation. Tension for change was universally reported to be high—respondents recognized the vital role of family engagement in the ICU but acknowledged that communication is challenging during restricted visitation. Respondents reported keen awareness of the negative impact of visitation restriction on family distress and patient personhood: “I would be terrified if I couldn’t visit my loved one in the ICU,” “I hope we can find a way to help patients feel human without their families at bedside.”

Compatibility, another implementation climate subconstruct, was also identified as a critical factor in the implementation of the family engagement navigator program. Both clinicians and navigators expressed concern about potential role confusion without explicit definition and awareness of navigator roles. Multiple respondents worried that families and navigators may experience frustration if navigators cannot provide clinical updates:

I wonder if this will stall if the [navigator] isn’t allowed to discuss medical issues. Most families want to know the day plans, test results, etc. They want to know what to expect—to do this adequately you have to give them some real information

Families might ask for information that cannot be shared by (the navigator), not understanding the role of the family navigator, not feeling completely satisfied with [navigator] communication compared to attending communication.

Accordingly, respondents expressed strong opinions that this intervention be additive not substitutive for communication with the clinical team.

Although respondents felt that the program could be a good fit into the existing operational workflow, they worried that poorly designed information exchange may result in inconsistent information provided to families and operational processes were viewed as necessary to promote consistency of communication with families. Respondents indicated that the inner setting culture would benefit program implementation because family engagement was an organizational priority even prior to COVID-19, and the participating ICUs had good track records of embracing new ideas.

### Domain 4: Individuals

Adequate knowledge about an intervention and positive attitudes toward it are important to adoption [17]. Although the family engagement navigators reported sufficient information about the program, some clinicians expressed a need for more information about the program. However, all respondents expressed strong value placed on family engagement, and most reported enthusiastic attitudes about its success. Self-efficacy is another key individual construct. Navigators reported confidence in their ability to deliver the family engagement support interventions and satisfaction with education and training resources.

### Domain 5: Process

Engagement is an important component of implementation success. Respondents indicated that the process of staffing the navigator workforce by self-selection was a major facilitator by ensuring that motivated and engaged individuals were involved in the program. Respondents also reported that another major facilitator stakeholder engagement was readily identifiable “champions,” or people in the organization who are likely to go above and beyond what might be expected, for the program [18]. A key construct of the implementation process is reflecting on and evaluating the progress of the implementation. Navigators expressed a desire to receive feedback from both families and bedside clinical teams to identify positive and negative implementation outcomes, and they indicated that receiving this feedback would benefit their engagement and motivation.

### Development of implementation strategies

Using contextual data obtained from step 1 and stakeholder feedback from step 2, we identified specific implementation strategies that are likely to be feasible and effective. These contextually appropriate implementation strategies are shown in Table 2, categorized by the ERIC taxonomy [15].

**Table 2:** Implementation strategies targeted to pre-implementation findings and categorized by the ERIC taxonomy [15]

	Application to the communication navigator program
Provide local technical assistance	Engage health information technology experts to assist if needed with communication resources. Enlist language services to provide support as needed.
Audit and provide feedback	Collect data and provide feedback to navigators and ICU clinicians on outcomes associated with the program including family experiences and outcomes. Regular debriefing meetings to enable identification and tackling of implementation challenges.
Develop educational materials/create online learning communities	Create educational resources and make them available for access online; documents amenable to continuous update as new knowledge and operations are available.
Conduct ongoing training	Navigators meet with a leader weekly to review cases and ensure procedures are being followed.
Promote adaptability	Clarify ways the navigator role can be tailored to meet individual needs and specify which elements of the innovation must be maintained to preserve fidelity.
Assess and redesign workflow	Monitor progress and adjust operational processes to continuously improve the efficiency and impact of the program. For example, we created an electronic health record note template to standardize information exchange (Supplemental File).
Identify and prepare champion	Reach out to respondents’ recommended champions and engage them with the program
Engage community resources	Organize community resources such as free phone and Internet access from local providers.

## Discussion

During this unprecedented pandemic, ICU leaders are charged to develop novel models of family engagement during restricted visitation. Our CFIR-informed rapid pre-implementation evaluation revealed several important insights that we used to inform our program and may help others design or adapt similar programs.

The use of the CFIR as an implementation framework aided in the analysis and interpretation of our data. The CFIR provided a practical framework for understanding stakeholder concerns, describing barriers and facilitators to implementation, and identifying appropriate next steps to implementation. Multilevel (organization, clinician, patient) processes were developed to support the implementation of the family engagement navigator. This pre-implementation assessment of the intervention characteristics, inner and outer settings, characteristics of individuals, and process provided a realistic assessment of feasibility, acceptability, and appropriateness of this model to be implemented in our ICU setting.

Our study is an exemplar of how implementation science methodology can be applied at a pace that allows efficient knowledge gain to inform program design under rapidly evolving time pressure such as the COVID-19 pandemic. Implementation science leaders have called for a novel methodology to speed the translation of knowledge to practice [8, 9]. This study demonstrates the feasibility of rapid execution of a pre-implementation evaluation that is conceptually based and explicitly focused on multiple implementation outcome dimensions relevant to program success. This foundational work allowed us to implement the program successfully and support 70 ICU patients’ families from May to July 2020 [19].

This study had several limitations. First, we used purposive sampling which is a non-probability sampling technique that could limit the representativeness of the sample. However, this a technique widely used in qualitative research for the selection of information-rich cases [20]. We stratified the sample by role to capture major variations in experience. Second, we used electronic surveys. Traditional in-person interviews may have provided additional information or richer detail, but we chose electronic format to maintain physical distancing and increase the efficiency of data collection in time to inform program design. Although videoconferencing interviews would have been appropriate, this study was conducted early in the pandemic when the study team was not yet comfortable using this approach for data collection. We purposely structured the survey to maximize responses >to open-ended questions and encouraged respondents to respond as if they were responding to an in-person interview. Notably, respondents provided rich and lengthy responses to the open-ended questions, suggesting the face validity of the approach. Rapid analysis approaches like this are gaining traction in implementation science and have been shown to produce valid findings in a short time frame [21]. Third, we only evaluated nurse, physician, and medical student input, and we were missing important insights from other relevant ICU stakeholders such as social workers, spiritual care providers, mental health therapists, and patients and families. Fourth, providing emotional support is a complex task, and medical students may require more extensive training than offered by this program [22]. Nonetheless, our rapid pre-implementation results found that medical students have self-efficacy for the emotional support tasks and felt that the training and available resources were adequate. Fifth, our study occurred at a single center, and our ICU may have different operational and clinical structures than other settings, which may limit generalizability. Future research will be needed to identify which contextual factors are associated with successful implementation outcomes of these novel programs. Finally, the sustainability of the program will be a major focus of future work, with maintaining the navigator workforce as a potential barrier [19]. Solutions could include leveraging the skill sets of additional multidisciplinary team members such as social workers, nurses or nursing students, and pastoral care providers either in a volunteer or paid position.

## Conclusions

Successful adoption of novel approaches to ICU family engagement may be improved with the integration of implementation science methods, including a rapid pre-implementation assessment to inform and adapt program design. We demonstrated successful application of a rapid methodologic approach that allowed efficient knowledge gain which can be a useful exemplar to others aiming to rapidly address new clinical challenges and incorporate new evidence in settings like the COVID-19 pandemic.

## Supplementary Information

**Additional file 1.** : Template for Intervention Description and Replication (TIDieR) checklist.

**Additional file 2.** : Family Engagement Navigator EHR note template.

## Data Availability

The datasets used and/or analyzed during the current study are available from the corresponding author on reasonable request.
