# Supplementary material for: A rapid pre-implementation evaluation to inform a family engagement navigator program during COVID-19
Source: Implement Sci Commun. 2020 Dec 9;1:110. doi: 10.1186/s43058-020-00098-2 (PMC7724442; doi:10.1186/s43058-020-00098-2)
Supplement: Supplementary file 1 — Additional file 1. : Template for Intervention Description and Replication (TIDieR) checklist. [file 43058_2020_98_MOESM1_ESM.pdf]

TIDieR

Family Engagement Navigator program

**Why:**	Innovative models of family engagement and support are needed in the intensive care unit (ICU) during times of restricted visitation such as the COVID-19 pandemic. One strategy involves leveraging skills of persons outside of the clinical team such as medical students, social workers, or providers in specialties other than critical care as “communication navigators” to provide additional support to families.2,5 This approach is supported by evidence for success of communication facilitators in other settings
**What (material):**	Medical students serving as Family Engagement Navigators received an initial training session including an "introduction to critical care and respiratory support interventions" lecture, empathetic listening modules (https://www.vitaltalk.org/topics/establish-rapport/ and https://www.vitaltalk.org/topics/track-respond-to-emotion/) and review of organizational visitation and communication policies.
**What (procedures):**	New ICU patients are assigned to navigators on the first day of admission. Navigators identify a primary contact for the family, help the family determine the best platform for communication (phone, videoconferencing application, etc.) and assist in overcoming any barriers to accessing communication platforms, including designating preferred timing of contact from clinical team. Navigators facilitate communication as needed, such as helping families keep a list of questions to ask the clinical team. Additionally, navigators provide emotional support to families, allowing them to process their fears, anxieties, and stress related to their loved ones’ illness.
**Who provided:**	The navigators are third and fourth year medical students who will have completed all preclinical and some clinical training. Navigators will have received the introductory training and have opportunities to ask questions or request additional training or support if needed.
**How (mode of delivery; individual or group):**	Navigators provide support via telephone or video-conference platforms, typically daily but the frequency of the calls are individualized to families' preference.
**Where:**	Navigators performed support from their homes. Navigator support required telephone access at minimum and free video conference software or applications for families open to video platforms.
**When and how much:**	Family Engagement Navigation was performed daily or more or less frequently per family preference. Navigation continued until patients were discharged from the ICU, expired, or family member expressed no longer wanting to receive support.
**Tailoring:**	Navigators tailor the frequency of contact and focus of support based on family needs.
**How well (planned):**	Navigators complete a "call log" in which they record length of calls, and note delivery of each of the intervention components at each call.
