# Supplementary material for: A rapid pre-implementation evaluation to inform a family engagement navigator program during COVID-19
Source: Implement Sci Commun. 2020 Dec 9;1:110. doi: 10.1186/s43058-020-00098-2 (PMC7724442; doi:10.1186/s43058-020-00098-2)
Supplement: Supplementary file 2 — Additional file 2. : Family Engagement Navigator EHR note template. [file 43058_2020_98_MOESM2_ESM.pdf]

Family Engagement Navigator EHR note template

Primary contact name:
Primary contact phone #:
Other family members:

Preferred time of day for contact:
Preferred contact platform (telephone, video app):

Today, family wishes to discuss:

"Get to know me" information
Patient likes to be called:
Favorites (if applicable):
  movie/tv show/book:
  music
  sports
  food
  pet
Activities/hobbies
Work/achievements
Things that stress patient out
Things that cheer patient up
Other things family would like you to know about patient
At home patient uses: (glasses, hearing aid, dentures, walking aid...)
